# Retinal vascular flow and choroidal thickness in eyes with early age-related macular degeneration with reticular pseudodrusen

**DOI:** 10.1186/s12886-018-0866-3

**Published:** 2018-07-28

**Authors:** So Min Ahn, Suk Yeon Lee, Soon-Young Hwang, Seong-Woo Kim, Jaeryung Oh, Cheolmin Yun

**Affiliations:** 1 Department of Ophthalmology, Korea University College of Medicine, 123, Jeokgeum-ro, Danwon-gu, Ansan-si, Gyeonggi-do, Seoul, South Korea; 2 Department of Biostatistics, Korea University College of Medicine, Seoul, South Korea

**Keywords:** Early age-related macular degeneration, Reticular pseudodrusen, Retinal atrophy, Optical coherence tomography angiography

## Abstract

**Background:**

To investigate the characteristics of retinal vessels and retinal thickness in eyes with early age-related macular degeneration (AMD) with or without reticular pseudodrusen.

**Methods:**

We retrospectively evaluated the clinical history and optical coherence tomography (OCT) and OCT angiography images of consecutive patients with early AMD. We calculated the retinal vessel densities of the superficial and deep capillary plexus with the ImageJ software (National Institutes of Health, Bethesda, MD, USA) and investigated the relationship with mean retinal thickness and subfoveal choroidal thickness.

**Results:**

We included 135 early AMD eyes and classified 60 of them into a reticular pseudodrusen group and 75 into a non-reticular pseudodrusen group. The vascular densities of the superficial and deep capillary plexus in the reticular pseudodrusen group (32.35% ± 3.67 and 26.71% ± 2.88%) were not different from those of the non-reticular pseudodrusen group (33.18% ± 2.2% and % 27.43 ± 1.79%; *P* = 0.546 and *P* = 0.318, respectively). The retinal thickness of the reticular pseudodrusen group (287.31 μm ± 24.36 μm) did not differ from that of the non-reticular pseudodrusen group (294.27 μm ± 20.71 μm; *P* = 0.493), while subfoveal choroidal thickness in the reticular pseudodrusen group (158.13 μm ± 42.53 μm) was lower than that in the non-reticular pseudodrusen group (237.89 μm ± 60.94 μm; *P* <  0.001). Multivariate analysis revealed that lower vascular density of the superficial capillary plexus and subfoveal choroidal thickness were associated with retinal thinning in reticular pseudodrusen group (*P* = 0.003 and *P* = 0.036) and older age was associated with retinal thickness in the non-reticular pseudodrusen group (*P* = 0.005).

**Conclusions:**

Retinal thinning in early AMD patients with reticular pseudodrusen was accompanied by choroidal and retinal vascular loss, which suggests a possible linkage of retinal thinning with vascular alterations.

**Electronic supplementary material:**

The online version of this article (10.1186/s12886-018-0866-3) contains supplementary material, which is available to authorized users.

## Background

Age-related macular degeneration (AMD) is a leading cause of legal blindness and its pathogenesis remains insufficiently understood [1]. Drusen are recognized as a hallmark in eyes with AMD and are considered to be risk factors for late AMD including neovascular AMD and geographic atrophy [1, 2]. However, with the development of ocular imaging techniques, an additional different phenotype, reticular pseudodrusen, has been identified and reported on. Reticular pseudodrusen, which was proposed by Mimoun et al., appears as a yellowish interlacing network on fundus examination and the appearance of subretinal drusenoid deposit located above the retinal pigment epithelium on optical coherence tomography (OCT) has been suggested to be associated with the development of late AMD [3–5].

Geographic atrophy is an advanced stage of late AMD characterized by the loss of the outer retina, retinal pigment epithelium, and underlying choriocapillaris [6]. Retinal atrophy in early AMD has been suggested to develop in retina-overlying drusen and these areas may subsequently progress to geographic atrophy [6–8]. However, recently, several studies have reported the distinct clinical features of retinal atrophy found in eyes with drusen versus in those with reticular pseudodrusen [6–11]. Eyes with geographic atrophy tend to have a higher prevalence of reticular pseudodrusen and their presence has been suggested to be associated with the progression of early AMD to geographic atrophy rather than to neovascular AMD [6, 12–15].

To date, the pathogenesis of reticular pseudodrusen is still not clear, but various imaging and histologic studies note that these eyes may have choroidal perfusion problems and suggest the existence of a possible association between such and a vascular basis [14, 16–20]. Recently, several studies performed involving OCT angiography (OCTA) revealed that early AMD eyes had significant retinal vascular alterations and that retinal vascular loss might be associated with retinal thinning in eyes with reticular pseudodrusen [21–23]. However, previous studies didn’t consider the effects of choroidal thickness as well as various factors including age, gender, and the presence of drusen that may affect the retinal thickness [24–28]. Because the choroid is composed of vessels and capillaries, choroidal thickness may represent the choroidal circulation and thinner choroid may be a marker of a damaged choroidal circulation which have insufficient blood flow to the choroid [29]. Several previous studies noted that abnormal choroidal circulation may be involved in the development of AMD [30, 31]. Therefore, in this study, we investigated the retinal vascular densities of the superficial capillary plexus and deep capillary plexus in early AMD with or without reticular pseudodrusen and their association with retinal thickness, considering choroidal thickness.

## Methods

This study was approved by the institutional review board of Korea University Medical Center in Seoul, Korea. All data collection and analysis efforts were conducted in accordance with the tenets of the Declaration of Helsinki.

We reviewed the medical records of early AMD patients who visited the clinic at Korea University Medical Center between June 2016 and January 2018 retrospectively. We defined early AMD cases as those eyes that demonstrated an early or intermediate stage of AMD according to the classifications of the Age-Related Eye Disease Study [32]. Presentations of late AMD included both neovascular AMD and geographic atrophy. All patients received comprehensive ophthalmic examinations including wide-field fundus photography, autofluorescence, and spectral domain OCT (SD-OCT) (Cirrus HD-OCT 5000; Carl Zeiss Meditec, Dublin, CA, USA.) and OCTA. We also collected information about systemic diseases, hypertension, and diabetes. We excluded any cases with a history of vitreoretinal surgery, vitreoretinal disease including diabetic retinopathy, epiretinal membrane, retinal vein occlusion, bilateral neovascular AMD, geographic atrophy, uveitis, and/or high myopia (axial length greater than 26.0 mm). In cases of bilateral early AMD patients, the right eye was chosen for analysis.

The reticular pseudodrusen area was defined as yellow interlacing network lesions ranging from 125 μm to 250 μm based on fundus examination and color fundus photography [18, 33]. The reticular lesions were identified on SD-OCT and defined when five or more hyperreflective triangular lesions or mounds are present above the retinal pigment epithelium in at least one of the B-scans in all images of the macular cube scans [34, 35]. Two independent observers (S.A. and C.Y.) classified AMD status and confirmed the existence of reticular pseudodrusen in each participant. In cases of disagreement, both observers reviewed the cases again and a final decision was made jointly. Data on the area of drusen under the retinal pigment epithelium in 3-mm- and 5-mm-diameter areas centered on the fovea were collected using advanced retinal pigment epithelium analysis using SD-OCT software [36]. The area provided by the software does not include the area of reticular pseudodrusen over the retinal pigment epithelium. The drusen area and volume were transformed to the square root value for statistical analysis [37].

The OCT device generated a volume scan with a 512 × 128 scan pattern and a line scan centered on the fovea with enhanced depth imaging protocol. Retinal thickness at the fovea and at four sectors of the 3 mm inner circle (i.e., the superior, nasal, inferior, and temporal) in the Early Treatment of the Diabetic Retinopathy Study chart were collected. Both observers (C.Y. and S.A.) reviewed all OCT B-scan images of volume scan and manually corrected segmentation errors together with assistance from the built-in OCT software in cases with segmentation errors.

Choroidal thickness was measured manually at the fovea using the line scan and a caliper tool integrated into the OCT software. The length was measured from the RPE to the inner surface of the sclera perpendicularly. Two retinal specialists (C.Y. and S.A.) performed independent measurements and the mean of the two measurements was used in the analysis.

The OCT device uses an 840 nm wavelength and an 68,000 A-scans/second speed with an OCT microangiography complex algorithm. The OCTA examination employed a 3 mm × 3 mm volume scan pattern centered on the fovea. We exported the en-face OCTA images of superficial capillary plexus and deep capillary plexus from the software. The superficial capillary plexus was segmented from the internal limiting membrane to the bottom of the inner nuclear layer, while the deep capillary plexus was segmented from the internal aspect of the inner nuclear layer to below the outer plexiform layer [38]. Each of the images were automatically segmented and both observers examined the segmentation errors. If segmentation errors are present, the observers corrected it together. We calculated the vascular density in the superficial capillary plexus and deep capillary plexus using the ImageJ software (version 1.49; National Institutes of Health, Bethesda, MD, USA) [39]. Initially, the en-face OCTA image was imported in the software. Then, the eight-bit image was processed with the command path Adjust > Auto local threshold with Otsu method > Process > Binary > Make binary > Edit > Selection > Create selection > Measure. Using the total number of pixels with vessels, the vascular densities of the superficial capillary plexus and deep capillary plexus were calculated as a ratio of the occupied area by vessels in the 3 × 3 area (number of pixels in the vessel area / number of pixels in total area × 100). The foveal avascular zone area of the superficial capillary plexus and deep capillary plexus was measured manually using the ImageJ software by two observers (C.Y. and S.A.). The mean foveal avascular zone area of the superficial capillary plexus and the deep capillary plexus was then calculated from the mean values obtained by the two observers and used in the analysis.

For image analysis, poor quality images (those with a signal strength of less than 7), OCTA images with motion artifacts extending over more than two lines, and those with vessel-duplication artifacts were excluded.

The normal distribution of continuous variables was determined with a Kolomogorov–Smirnov test. A comparison of variables between the two groups was performed with an independent t-test or Mann–Whitney U test for continuous variables and a chi-squared test for the categorical variables. To adjust for the differences of age between the two groups, the analysis of covariance (ANCOVA) test was applied. Pearson’s correlation or Spearman’s rank test was used to analyze the relationship between continuous variables. Simple linear regression was used to analyze the relationship of various parameters and the RT. Based on the results of simple linear regression, we included variables with statistical significance for the multiple linear regression and determined significant factors with backward elimination. Inter-observer reliability was assessed with an intraclass correlation coefficient. Statistical analysis was performed with the SPSS software (version 20.0 for Windows; IBM Corporation, Armonk, NY, USA). *P*-values < 0.05 were considered to be statistically significant. In cases of comparisons, because there were multiple comparisons for outcome parameters between two groups, we used an adjusted P-value with the Bonferroni correction.

## Results

The reticular pseudodrusen group and non- reticular pseudodrusen group included 60 eyes and 75 eyes, respectively. The mean age (years) of the patients in the reticular pseudodrusen group was greater than that of those in the non- reticular pseudodrusen group (*P* = 0.001). The gender, history of hypertension, diabetes, and mean axial length were not different between the two groups (Table 1). The mean subfoveal choroidal thicknesses of the two groups were different (*P* <  0.001) and the square root of the 5 mm drusen area of the reticular pseudodrusen group was greater than that of the non- reticular pseudodrusen group (*P* = 0.045). Conversely, the mean retinal thickness and square root of the 3 mm drusen area were not different between the two groups (*P* = 0.075 and *P* = 0.071). After adjustment for age, the square root of the 5 mm drusen area was not different between the two groups (*P* = 0.434) (Table 2). In each group, 16 patients in the reticular pseudodrusen group and 24 patients in the non-reticular pseudodrusen group had late AMD in one eye.

**Table 1:** Comparison of baseline characteristics between early AMD patients with and without reticular pseudodrusen

	Reticular pseudodrusen group (*n* = 60)	Non-reticular pseudodrusen group (*n* = 75)	*P*-value
Age (years)	74.77 ± 8.70	68.76 ± 10.97	0.001*
Gender (male:female)	14:46	26:49	0.152^†^
Hypertension, n (%)	28 (46.7%)	36 (48.0%)	0.728*
Diabetes, n (%)	17 (28.3%)	22 (29.3%)	0.899*
Axial length (mm)	23.38 ± 1.23	23.11 ± 1.01	0.223*

*Independent t-test

^†^Chi-square test

**Table 2:** Comparison of baseline optical coherence tomographic characteristics between early AMD patients with and without reticular pseudodrusen

	Reticular pseudodrusen group (*n* = 60)	Non-reticular pseudodrusen group (*n* = 75)	*P*-value*	Age-adjusted *P*-value^†^
Mean retinal thickness (μm)	287.31 ± 24.36	294.27 ± 20.71	0.075	0.493
Subfoveal choroidal thickness (μm)	158.13 ± 42.53	237.89 ± 60.94	< 0.001	< 0.001
3 mm drusen area (mm^2^)**	0.50 ± 0.74	0.37 ± 0.73	0.308	0.929
5 mm drusen area (mm^2^)**	0.80 ± 1.07	0.55 ± 1.02	0.182	0.764
Square root of 3 mm drusen area	0.50 ± 0.51	0.33 ± 0.51	0.071	0.519
Square root of 5 mm drusen area	0.65 ± 0.62	0.44 ± 0.61	0.045	0.434

*Independent t-test, P-value < 0.005 (0.05/10) was considered to be statistically significant with the Bonferroni correction

^†^ANCOVA test, P-value < 0.005 (0.05/10) was considered to be statistically significant with the Bonferroni correction

**Drusen area under the retinal pigment epithelium

The mean foveal avascular zone areas of the superficial capillary plexus and deep capillary plexus of the reticular pseudodrusen group were not different from those of the non- reticular pseudodrusen group (*P* = 0.734 and *P* = 0.594). The mean vascular densities of the superficial capillary plexus and deep capillary plexus of the reticular pseudodrusen group also did not show differences in comparison with those of the non- reticular pseudodrusen group (*P* = 0.106 and *P* = 0.089) (Table 3).

**Table 3:** Comparison of angiographic features between early AMD patients with and without reticular pseudodrusen

	Reticular pseudodrusen group (*n* = 60)	Non-reticular pseudodrusen group (*n* = 75)	*P*-value*	Age-adjusted *P*-value^†^
Superficial capillary plexus				
Foveal avascular zone area (mm^2^)	0.34 ± 0.11	0.34 ± 0.13	0.734	0.553
Vascular density (%)	32.35 ± 3.67	33.18 ± 2.22	0.106	0.615
Deep capillary plexus				
Foveal avascular zone area (mm^2^)	1.28 ± 3.78	1.31 ± 3.80	0.594	0.322
Vascular density (%)	26.71 ± 2.88	27.43 ± 1.79	0.089	0.352

*Independent t-test, P-value < 0.005 (0.05/10) was considered to be statistically significant with the Bonferroni correction

^†^ANCOVA test, P-value < 0.005 (0.05/10) was considered to be statistically significant with the Bonferroni correction

**Fig. 1:**
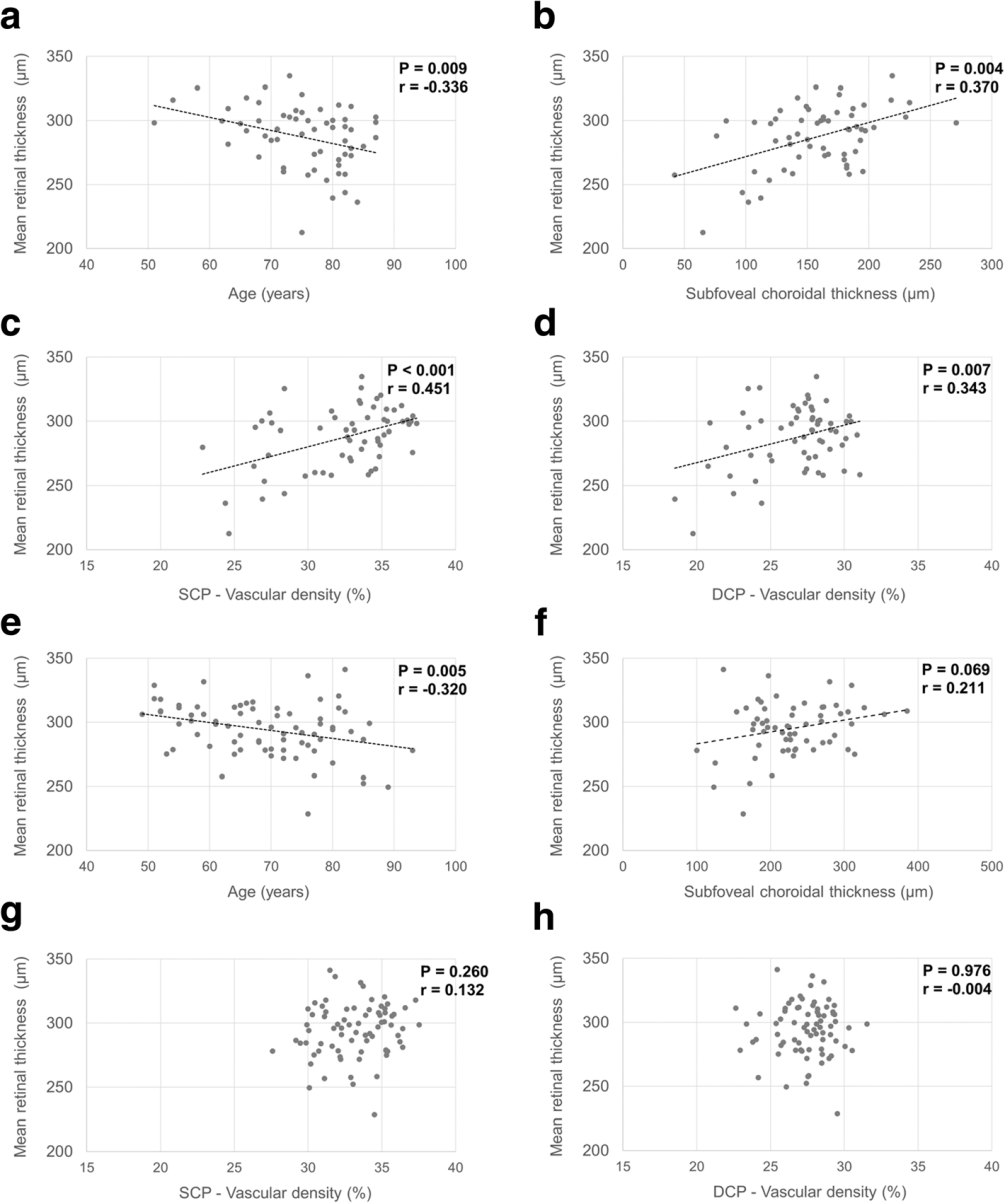
Scatter plots of retinal thickness and its associations with age, subfoveal choroidal thickness, and vascular densities. In the reticular pseudodrusen group (**a-d**), retinal thickness was negatively associated with age and the vascular densities of the superficial capillary plexus and deep capillary plexus and was positively associated with subfoveal choroidal thickness. In the non- reticular pseudodrusen group (**e-h**), retinal thickness was negatively associated with age and positively associated with subfoveal choroidal thickness and had no relationship with the vascular densities of the superficial capillary plexus and deep capillary plexus

**Fig. 2:**
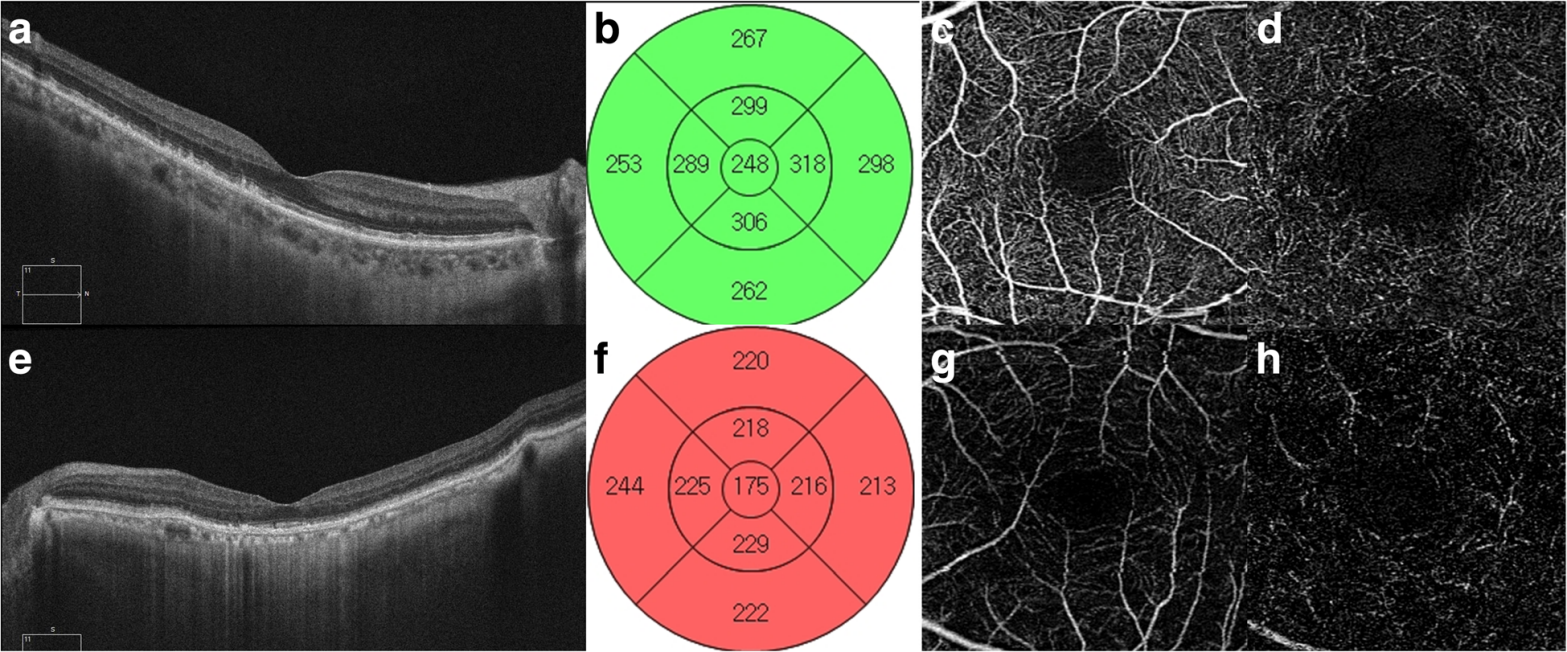
Representative cases of patients with early AMD and reticular pseudodrusen. OCT and OCTA images of a 71-year-old female patient (**a-d**) and a 75-year-old male patient (**e-h**) with early AMD and reticular pseudodrusen. **a** Line scan shows multiple subretinal drusenoid deposits with 193 μm subfoveal choroidal thickness. **b** ETDRS grid shows normal range retinal thickness. **c** and **d** OCTA shows relatively preserved superficial capillary plexuses and deep capillary plexuses. **e** Line scan shows multiple subretinal drusenoid deposits with 65 μm subfoveal choroidal thickness. **f** ETDRS grid shows decreased retinal thickness. **g** and **h** OCTA shows relatively decreased vascular densities of the superficial capillary plexus and deep capillary plexus

In the reticular pseudodrusen group, the mean retinal thickness was associated with age, subfoveal choroidal thickness, square root of the 5 mm drusen area, and vessel density of the superficial capillary plexus and deep capillary plexus (Fig. 1 and Table 4). In the non- reticular pseudodrusen group, the mean retinal thickness was associated with age, subfoveal choroidal thickness, and the square root of the 5 mm drusen area (Fig. 1 and Table 5). In multivariate analysis, a thinner subfoveal choroidal thickness and a lower vessel density of the superficial capillary plexus were associated with lower retinal thickness in the reticular pseudodrusen group (Table 6), while older age was associated with lower retinal thickness in the non- reticular pseudodrusen group. Representative cases are presented in Fig. 2.

**Table 4:** Univariate analysis for estimating factors associated with mean retinal thickness in the reticular pseudodrusen group

Variables	ß	Standard error	*P*-value*
Age (years)	−1.020	0.376	0.009
Gender (female)	11.510	7.344	0.122
Hypertension	−5.331	6.336	0.404
Diabetes	5.888	6.995	0.403
Axial length (mm)	−3.353	3.036	0.275
Subfoveal choroidal thickness (μm)	0.212	0.070	0.004
Square root of the 3 mm drusen area^†^	−10.090	6.160	0.107
Square root of the 5 mm drusen area^†^	−8.991	5.049	0.080
Foveal avascular zone area of superficial capillary plexus (mm^2^)	−26.571	28.805	0.360
Foveal avascular zone area of deep capillary plexus (mm^2^)	4.938	8.437	0.561
Vessel density of superficial capillary plexus (%)	2.995	0.778	< 0.001
Vessel density of deep capillary plexus (%)	2.900	1.044	0.007

*Simple linear regression

^†^Drusen area under the retinal pigment epithelium

**Table 5:** Univariate analysis for estimating factors associated with mean retinal thickness in the non- reticular pseudodrusen group

Variables	ß	Standard error	*P*-value*
Age (years)	−0.622	0.215	0.005
Gender (female)	−2.716	5.049	0.592
Hypertension	−6.025	4.767	0.210
Diabetes	−5.711	5.246	0.280
Axial length (mm)	4.010	2.813	0.160
Subfoveal choroidal thickness (μm)	0.072	0.039	0.069
Square root of 3 mm drusen area^†^	−7.404	3.255	0.118
Square root of 5 mm drusen area^†^	−6.723	2.321	0.089
Foveal avascular zone area of superficial capillary plexus (mm^2^)	−14.296	19.013	0.455
Foveal avascular zone area of deep capillary plexus (mm^2^)	−0.161	6.393	0.980
Vessel density of superficial capillary plexus (%)	1.229	1.084	0.260
Vessel density of deep capillary plexus (%)	−0.041	1.354	0.976

*Simple linear regression

^†^Drusen area under the retinal pigment epithelium

**Table 6:** Multivariate analysis for estimating factors associated with mean retinal thickness in the reticular pseudodrusen group

Variables	ß	Standard error	*P* value*
Vessel density of superficial capillary plexus (%)	2.475	0.792	0.003
Subfoveal choroidal thickness (μm)	0.147	0.068	0.036

*Multiple linear regression

We assessed the interobserver reproducibility for the subfoveal choroidal thickness and foveal avascular zone area of the superficial capillary plexus and deep capillary plexus with an intraclass correlation coefficient. The intraclass correlation coefficient was 0.895 [95% confidence interval (CI): 0.852–0.925] for subfoveal choroidal thickness; 0.931 (95% CI: 0.903–0.951) for the foveal avascular zone of the superficial capillary plexus; and 0.861 (95% CI: 0.804–0.901) for the foveal avascular zone of the deep capillary plexus, respectively.

## Discussion

Recently, several studies have suggested that vascular alterations are involved in the pathogenesis of AMD [1, 6, 7, 22, 23, 30, 31]. Diminished vascular flow in the retina and choroid has been considered as a factor associated with AMD development and progression [30, 31]. In the current study, the mean vascular density of the superficial and deep capillary plexus were not different between in the reticular pseudodrusen group versus in the non-reticular pseudodrusen group after adjustment for age. In the reticular pseudodrusen group, thinner choroidal thickness and lower retinal vascular density were associated with retinal thinning, while age was associated with retinal thickness in the non-reticular pseudodrusen group.

Previous studies have suggested that eyes with early AMD demonstrate significant changes in retinal vascular flow and that such was more pronounced in eyes with reticular pseudodrusen [21–23]. Toto et al. reported the occurrence of retinal vascular impairment and associated retinal damage in patients with early AMD [21, 22]. However, because they did not consider the presence of reticular pseudodrusen, the impact of reticular pseudodrusen on vascular flow was not suggested. More recently, Cicinelli et al. investigated the retinal vessels according to the presence of reticular pseudodrusen on OCTA and suggested that retinal vascular loss is present in early AMD patients and that these features are more pronounced in eyes with reticular pseudodrusen [23]. These reports suggested that retinal thickness is reduced with changes in retinal vascular loss. However, retinal thickness has been reported to be affected by various factors including age, gender, spherical equivalent, and drusen [8, 27, 28, 40, 41]. In addition, alterations in choroidal thickness are accompanied by retinal vascular changes in early AMD patients [21]. However, these previous studies did not consider these factors in the context of association with retinal thickness. Therefore, we included these factors in the analysis in the present study. The results of our study are similar to those from previous studies. However, we found additional factors that might be associated with retinal thickness in eyes with early AMD in addition to the retinal vascular changes.

In this study, in spite of the similarity of retinal thickness and vascular densities of superficial and deep capillary plexus, different associations of retinal thickness and vascular density were observed between the two groups. This suggests that development of retinal thinning in early AMD eyes may have different features of retinal vasculature according to the presence of reticular pseudodrusen. Previous studies suggested that ocular perfusion is decreased in several ocular diseases and correlated with the degree of the severity or damage of neural retina [26, 42, 43]. Retinal thickness has a correlation with the retinal vascular perfusion and the decreased retinal vascular flow might reflect the status of damaged retina [44]. Drusen-related retinal atrophy usually develops on the area overlying the drusen and after drusen regression [7]. It is confined to areas affected by the drusen and thus, the retinal degenerative changes are focal rather than overall and the changes may not induce whole macular changes. Thus, retinal thickness measurements might not reflect the whole retinal change in the non- reticular pseudodrusen group in this study. However, in eyes with reticular pseudodrusen, diffuse changes in retinal thickness which were associated with retinal and choroidal vascular changes were noted. Because the reticular pseudodrusen group had similar drusen characteristics to those of the non- reticular pseudodrusen group, there might be another factor that may affect the retinal changes. Previous studies suggested that the eyes with reticular pseudodrusen are under diffuse choroidal changes and the etiology of which was suggested to be a vascular problem by comparison with eyes without pseudodrusen [14, 16, 23, 45–47]. In addition, the characteristics of retinal atrophy in eyes with AMD were reported to be different between eyes with and without reticular pseudodrusen, and the eyes with reticular pseudodrusen have a tendency to have diffuse and multilobular retinal atrophy [48]. In conjunction with previous reports and our study, diffuse changes in retina and choroid are present in eyes with reticular pseudodrusen compared to those without reticular pseudodrusen, and this might be associated with retinal thinning on a vascular basis.

In the non-reticular pseudodrusen group, lower retinal thickness was associated with older age, a larger area of drusen, and a thinner subfoveal choroidal thickness. Because the square root of drusen area and subfoveal choroidal thickness had a relationship with age in this study, their effects on retinal thickness became attenuated with multivariate analysis (See Additional file 1). Usually, it has been suggested that the amount of drusen increase over time in early AMD eyes and that the choroid undergoes atrophy with advancements in age [25, 37]. Thus, even though various factors were associated with retinal thinning in the non- reticular pseudodrusen group, retinal thickness in early AMD eyes without reticular pseudodrusen might be predominantly influenced by aging. However, in the reticular pseudodrusen group, there might be other mechanisms that affect retinal thickness. Several investigators including individuals from our group previously reported about decreased choroidal thickness in early AMD eyes with reticular pseudodrusen when they were compared with early AMD eyes without reticular pseudodrusen [26, 45–47]. In addition to the finding of decreased choroidal thickness, we also suggested that the progression of choroidal atrophy is more prominent in eyes with reticular pseudodrusen and hypothesized that eyes with reticular pseudodrusen have an altered choroid that cannot compensate for changes in perfusion pressure appropriately, and that this might be associated with the prominent choroidal atrophy seen in eyes with reticular pseudodrusen [26]. Because the choroid supplies blood to the outer retina, changes in the choroid might therefore affect retinal status. The different features between the two groups in our study supports previous theories that have suggested that early AMD eyes with reticular pseudodrusen might be distinctly different from early AMD eyes without reticular pseudodrusen [7, 10, 17, 48–52].

We can suggest two possibilities with respect to the results of this study. First, the eyes with reticular pseudodrusen under chronic choroidal insufficiency might contribute to retinal atrophy [11, 20, 53]. It is well-known that retinal atrophy is common in eyes with reticular pseudodrusen [9, 11]. Decreased metabolic demand associated with outer retinal atrophy might induce secondary decreased retinal vascular flow and this means that choroidal changes might also contribute to secondary retinal change. Second, eyes with reticular pseudodrusen may be co-morbid for choroidal insufficiency and retinal vascular dysregulation. The regulation of blood flow to the retina and the choroid is quite different: while retinal flow vasculature employs autoregulation, choroidal flow employs autonomic regulation [42–44]. Although the exact mechanism of autoregulation of the retinal blood flow is unclear, several studies have shown that retinal blood flow responds to changes in ocular perfusion pressure and attempts to maintain a constant blood flow [42–44]. However, previous research has also reported that retinal vascular reactivity might decrease with age [42–44]. We hypothesized that eyes with reticular pseudodrusen may have a limited compliance with changes in ocular perfusion pressure both in choroid and retinal vessels, while the choroid and retinal vessels in early AMD eyes without reticular pseudodrusen might compensate better for changes in ocular perfusion pressure [26]. The contribution of decreased retinal vascular density independently with the choroidal thickness in this study might support this possibility. The etiology of reticular macular disease is controversial, but there are some findings that support the existence of a vascular basis with the alteration of choroid and choriocapillaris blood flow [14, 16, 54]. Smith et al. previously reported about the relationship between various systemic diseases that may affect systemic circulatory status and reticular macular disease [13, 16, 18]. They suggested a hypothesis that the impairment of blood flow is involved in the pathogenesis of reticular macular disease [14, 16, 54]. Retinal vascular changes including generalized narrowing of arteriole and venule has been recently reported to be associated with cardiovascular disease [55, 56]. In addition to the suggestions of previous investigations, the results of our study may give rise to a suspicion about the role of vascular origin in retinal atrophy in eyes with reticular pseudodrusen.

This study has several limitations. First, it has a retrospective design and includes a limited number of cases. There might be selection bias due to hospital-based sampling. Second, we diagnosed and classified patients into two groups based only on fundus examination and OCT. Other multimodal imaging options including infrared imaging might improve the diagnostic rate, even though reticular pseudodrusen can be detected with higher sensitivity and specificity only with OCT [34]. Third, because reticular pseudodrusen patients have characteristics associated with older age, which also have been reported in previous epidemiologic studies, we had to adjust the age with the statistical method [13, 35, 53]. Fourth, because of the limited scan area of OCTA, we investigated only in 3 mm × 3 mm area. Fifth, because we used SD-OCT with limited resolution on choriocapillaris, we could not investigate the OCTA images of choriocapillaris [57, 58].

In conclusion, patients with early AMD with reticular pseudodrusen showed retinal thinning accompanied by choroidal and retinal vascular loss, while patients without reticular pseudodrusen did not. This provides a suggestion that progression of retinal thinning in eyes with reticular pseudodrusen may be occur a vascular basis.

## Additional file

Additional file 1: **Table S1.** Relationship of retinal and choroidal parameters with age in the RPD and non-RPD groups. (DOCX 30 kb)

## Abbreviations

AMD: Age-related macular degeneration
OCT: Optical coherence tomography
OCTA: Optical coherence tomography angiography
SD-OCT: Spectral domain optical coherence tomography
