# Supplementary material for: Retinal vascular flow and choroidal thickness in eyes with early age-related macular degeneration with reticular pseudodrusen
Source: BMC Ophthalmol. 2018 Jul 28;18:184. doi: 10.1186/s12886-018-0866-3 (PMC6064139; doi:10.1186/s12886-018-0866-3)
Supplement: Supplementary file 1 — Table S1. Relationship of retinal and choroidal parameters with age in the RPD and non-RPD groups. (DOCX 30 kb) [file 12886_2018_866_MOESM1_ESM.docx]

Additional file 1: Table S1. Relationship of retinal and choroidal parameters with age in the RPD and non-RPD groups.

|  | RPD group | | Non-RPD group | |
| --- | --- | --- | --- | --- |
|  | P-value* | r | P-value* | r |
| Subfoveal CT (µm) | 0.015 | -0.313 | < 0.001 | -0.793 |
| Mean RT (µm) | 0.009 | -0.336 | 0.005 | -0.320 |
| Square root of 3 mm drusen area | 0.007 | 0.346 | 0.002 | 0.346 |
| Square root of 5 mm drusen area | 0.008 | 0.338 | 0.001 | 0.386 |
| FAZ area of SCP (mm^2^) | 0.208 | 0.165 | 0.782 | 0.032 |
| FAZ area of DCP (mm^2^) | 0.208 | 0.165 | 0.293 | 0.123 |
| Vessel density of SCP (%) | 0.003 | -0.382 | 0.015 | -0.280 |
| Vessel density of DCP (%) | 0.020 | -0.299 | 0.087 | -0.199 |

*Pearson’s correlation test.

CT, choroidal thickness; RT, retinal thickness; FAZ, foveal avascular zone; SCP, superficial capillary plexus; DCP, deep capillary plexus; RPD, reticular pseudodrusen.

.
